# Progress in Surgery

**Published:** 1899-06-03

**Authors:** 


### Progress in Surgery.

### HERNIA.

Dr. Braisted,[1] in a paper on Inguinal Hernia, discusses the very important question of the advisability of recommending the victims of this trouble to submit to operation for its cure, especially from the point of view of the army and navy. In European services, where military life is compulsory, it seems that at least 20 per cent. of men suitable for admission are considered to be incapacitated because they have inguinal hernia. Soldiers and sailors are often required of necessity to undertake sudden exertion, which is likely to produce hernia if there is a predisposition thereto. The perpetual gymnastics in ship life are specially provocative. If an officer or man does develop a hernia he is always in dread of being called upon to make special exertions, and the fear of incapacity at a critical moment haunts him. A brilliant career may thus be abruptly ended, and valiant and trained men be lost to the service. Do the possibilities of operative surgery afford ground to think that any considerable proportion of the men who acquire this affliction in the services may be cured so effectually as to render their retention in the service advisable?

This writer approves of the recently-adopted regulations in the United States army which provide that the men shall be offered the opportunity of having a radical cure performed, and advocates the adoption of similar regulations in the navy. The methods of cure are then discussed, and two methods specially approved, viz., the open method of McBurney, and the closed method of Bassini or Halsted. Of the former he reports 19 operations since 1887, all permanently cured, no truss being required, and says it is the safest and easiest method, and especially adapted for emergency cases where asepticism cannot be absolutely assured, where septic conditions already exist, and in any strangulated hernia where there is uncertainty as to the future recovery of the intestine. McBurney himself, however, now usually performs Bassini's operation. It may be here noted that Keen and White, in their "Text-book of Surgery," assert that they cannot find impartial operators of distinction assured of success in more than 50 per cent. of their cases. Ferguson[2] says it is not probable that we can at present properly urge the possessor of a hernia which is comfortably retained by a truss to submit to an operation; but points out that there is a tendency for hernia contracted in early adult life to become direct in their course, with enlarging rings, and retentive apparatus then becomes less efficient.

**The Technique in Radical Cure of Inguinal Hernia.—** There is a most extraordinary diversity of opinion upon the value of the different methods of performing radical cure. With regard to treatment of the sac, for example, Dr. Hopkins[2] asserts that it is certainly a mistake to displace the divided end (neck) of the sac from its normal position, and that the ligated and puckered stump is of great service in preventing the descent of the hernia, if it is fixed over the line of union of the internal ring, for it places a resistant mass of solid tissue in that position. Wheeler,[3] on the other hand, says: "Every one who has studied the recurrences of hernia will agree with the writers who state that consequent upon the ligation of the neck of a hernial sac a depression must exist, and this in turn will most likely enlarge and become infundibuliform." Such an infundibuliform condition of the peritoneum seems to offer a special inducement to the return of the hernia. The same writer says he has known death follow a promised radical cure by twisting of the sac; that he has himself never twisted a sac, and considers it unscientific surgery. Attention has been drawn by Lynn Thomas[4] to a new method of displacement of the sac, introduced by Kocher, which seems likely to be very successful. The end of the sac is seized in a special forceps, and inverted into the peritoneal cavity, and brought out through the anterior abdominal wall nearly opposite the anterior superior iliac spine, made taut there, and fixed to the parietal peritoneum and transversalis fascia. The projecting portion is cut off, and the small wound in the muscles sutured. Being inverted it is the peritoneal surface of the sac which is seen at the point of extrusion through the parietes. A similar mode of displacement is available in femoral hernias.

With regard to the management of the spermatic cord Mr. McArdle[5] writes, "There is no evidence in favour of opposing the arrangements of nature by displacing the cord, as in Bassini's method." G. G. Hopkins[6] also writes, "I doubt very much whether anything is gained by the displacement of the cord, as suggested by Bassini, and now almost universally practised. When we try to improve upon Nature we generally go astray." He regards it as bad in principle, also because the displacement upwards offers an increased purchase for the downward abdominal pressure to bear upon. Owing to this pressure the cord will exert special tension upon the line of sutures closing the internal ring, which it would not do if brought out at the lower part of the canal.

In closing the canal and rings, Ferguson[2] alludes to the readiness with which muscle fibres can be separated longitudinally, as showing that the protection which they will afford alone against the protrusion of a hernia is slight. The main factor in protecting against hernia is dense aponeurosis or fascia, and he considers that no scar exercises the protective influence of normal fascia. In this connection McArdle's plan of suturing the lower flap of the external oblique muscle, under the upper, and bringing down the latter over it to be fixed to the deep fascia of the thigh below Poupart's ligament is worthy attention.

As suture material in these operations catgut, silk, kangaroo tendon, and silkworm gut each have their advocates. Silk, though in the hands of many it is found satisfactory, yet with others has frequently led to reopening of the wound months after the operation for the discharge of sutures. Kangaroo tendon is thought to be best by many (*e.g.*, Langton[7]). This writer asserts that the proportion of suppurative cases at St. Bartholomew's amounted to 16·5 per cent., and attributes it mainly to insufficient cleansing of the skin. Most surgeons are of opinion that the complication lessens the full benefit of the operation. Stanley Boyd[7] confesses to having had some suppuration, and observes that there is no operation in which suppuration gives so much trouble. He thought that the best way to deal with it was to give an anæsthetic and take out some of the external stitches, and pass a tube through the internal oblique and transversalis to near the neck of the sac. Boyd applies torsion to the sac. In one case the bladder was unfortunately included in the ligature with a fatal result.

Langton does not care to operate upon patients under six years of age, but Owen and Boyd regard three years of age as the limit, as children of that age have acquired control of the bladder.

Langton states that 80 per cent. of private cases were successful. The records of the London Truss Society showed that in the last six years 242 patients were treated on whom operations had been performed but had failed to cure. He believed it to be better to wear a truss for a time after the operation.

Braisted (*op. cit.*) notices, though not with marked approval, a method of displacement of the spermatic cord introduced by Drs. Nélaton and Ombredanne. They divert the proximal end of the spermatic cord from the inguinal canal and cause it to run through a hole made in the os pubis. They can then close the canal more effectually than in Bassini's method. A button of bone is removed from the front of the pubic bone with a punch. The bridge of bone above this is divided by a chain saw, and the portion of bone raised up to lift the cord into the hole, and then replaced, and the abdominal wall repaired in two layers. This is obviously a severer operation than most surgeons would care to perform, unless there was very strong evidence of its exceptional effectiveness. It seems to be very rare in the practice of most to cause any permanent injury to the spermatic cord by the transplantation of the Bassini method. With the cord passing through a newly-formed bony ring, trouble seems very likely to arise from compression.

[1] Med. Rec., Jan., 1899. [2] Med. Rec., Jan., 1899. [2] Med. Rec., Nov., 1898. [3] Brit. Med. Jour., Nov. 5, 1898. [4] Bristol Med. Chi. Jour., Dec., 1898. [5] Dublin Jour. of Med. Sci., Dec., 1898. [6] Op. cit. [7] Lancet, Mar., 1899.

---

### GYNÆCOLOGY.

(*Continued from page* 148.)

**Septic Infection of Ovarian Cysts.** — Cumston,[9] of Boston, says that inoculation experiments have shown that if the liquid contents of an ovarian cyst were not contaminated by bacteria they would remain aseptic, and would undergo no change; but if, on the contrary, microbes attacked a cyst and entered it, its contents would serve as excellent culture media and symptoms of infection would soon appear. There are two kinds of septic infection of ovarian cysts, namely, pathogenic infection and saprophytic infection. Formerly pathogenic and saprophytic organisms often entered ovarian cysts through puncture, incision, and drainage; but as these methods of treating ovarian cysts are now practically discarded, it is clear that the patient contains the agent of the septic process under consideration. The germs did not come from without, and the process might be called a true auto-infection, which took place in one of three ways: (1) By means of the blood, in which case the infection was direct, produced by phlebitis, which extended up to the cyst; or indirect, in which case the infective elements were carried in the general circulation and reached the tumour by means of its pedicle; (2) by way of the lymphatics, in which case the lymphatic channels allowed a direct introduction of the germs into the interior of the cyst through its hilum; and (3) through adhesions which were plentifully supplied with new-formed vessels intimately connected with the vessels in the wall of the cyst, thus allowing an easy transportation of bacteria. After giving at considerable length the symptoms of ovarian cysts, and dwelling upon the diagnosis and differential diagnosis, the author deals with their operative treatment. Operation for the removal of ovarian cysts which are the seat of septic infection may be divided into four stages. The first is incision of the abdomen; the second, the breaking down of adhesions and ligation of blood vessels which they contain; the pus should then be removed by the trocar, because if the cyst was incised with the knife pus would immediately flow out and the wound very likely become infected by the septic material. The third step is the extraction of the cyst through the wound, and in doing this the surgeon must be careful to avoid infecting the abdominal incision in drawing the cyst through. This complication can be easily avoided if aseptic gauze sponges are lightly packed around and inside the line of incision; the pedicle is then ligatured and the stump dropped back into the abdomen. The fourth step consists in the cleansing of the peritoneum, which must be done with great care, especially when the operative field has run any chance of infection. If this has occurred free irrigation of the peritoneum is advisable, but it should be done with care, and the liquid employed should be a warm decinormal salt solution. Care should be taken to limit the irrigation to the sub-umbilical portion of the peritoneal cavity, and a back flow of the liquid towards the diaphragm prevented by having the operating-table perfectly flat and the thorax slightly raised.

**Malignant Disease of the Cervix Uteri.**—F. J. McCann[10] describes six cases illustrating the varieties of malignant disease met with in the neck of the womb, and treated by vaginal hysterectomy. He remarks that it is difficult, and in many cases impossible, to state the actual duration of the disease. Most cases have at first no symptoms at all, or the symptoms are so slight as to attract little attention. The sufferer does not seek advice, therefore, until the disease is far advanced. The earliest and most frequent sign of malignant disease is the hæmorrhage. This may occur after sexual intercourse, or it may be evidenced by increased bleeding at the menstrual periods; and loss of blood between the periods is common. When the discharge from the vagina has become foul the smell will indicate that sloughing of the diseased surface has commenced; this is, therefore, a later sign. Pain is a late symptom of cancerous disease, yet it is all-important to the patient, for unless pain exists many patients do not seek advice, hence the reason why cases are so frequently discovered when the disease cannot be removed. When pain exists of a severe character, interfering with sleep and requiring narcotics for its relief, the disease will be found to have spread to the pelvic cellular tissue and peritoneum. McCann urges, therefore, that every patient who has had a loss of blood from the vagina after the cessation of the menstrual periods should be carefully examined, as this hæmorrhage may be the initial sign of malignant disease. Further, enlargement of the uterus after the menopause should always give rise to suspicion. As to the causation of cancer, he thinks it may yet prove to be a parasitic disease, and he is a firm believer in the importance of gonorrhœal infection as a predisposing cause of cancer of the cervix, more especially when that infection leads to a chronic cervical gleet. Lacerations of the cervix are important, not because they are the future seat of malignant degeneration, but because they are accompanied by thickening and enlargement of the cervix and body of the uterus from the absorption of septic products, and thus predispose the tissue to malignant degeneration. Indeed, the septic subinvoluted uterus pouring forth a copious discharge from its cavity is, he thinks, a most important factor in the causation of cancer.

**A New Method of Treatment for Prolapse of the Uterus.**—Inglis Parsons[11] points out that there appear to be three main causes of prolapsus uteri: (1) Failure of the uterine ligaments; (2) laceration, weakness, and relaxation of the pelvic floor; (3) increase in weight of the uterus; and he describes a method of treatment which aims at strengthening the broad ligaments by exciting an effusion of lymph in their structure, by the injection of a suitable fluid in the cellular tissue of the broad ligaments, and he gives notes of ten cases in which he successfully adopted this method. The patient is placed in the lithotomy position, the vagina is thoroughly douched and rendered as aseptic as possible; the perineum is then retracted with either Auvard's or Sim's speculum, another retractor is used to hold up the anterior vaginal wall if necessary, and the lateral walls are thus exposed. The uterus is held with one hand as nearly as possible in its normal position by a probe passed into its interior; with the other hand the injections are made on each side through the vaginal wall into both broad ligaments on a level with the external os, and from about three-quarters of an inch to an inch from the junction of the vagina with the cervix. The fluid used for injection was a solution of quinine; after the syringe and speculum have been withdrawn it is necessary to keep the uterus up with a cup and stem pessary held in position by four tapes tied round the waist; this is left in position for three or four days, and is then removed. A special syringe with a long thin nozzle is a great convenience, because it does not obstruct the light, and it enables the operator to see that the needle point pierces the vaginal wall at the right spot. When the quinine solution was injected it sometimes caused no pain; in other cases slight pain was experienced. After the operation most of the patients had practically no pain. The amount of effusion will depend on the personal reaction of the individual, and the strength and amount of the solution injected. The resulting swelling is not hard like a parametritis, nor is it at all extensive, but feels more like a band, in most cases passing from the uterus to the wall of the pelvis; it is firm and resistent, and the uterus while being held still retains its mobility.

**Uterine Hæmorrhage as Affected by the Climate of Altitudes.**—Septimus Sunderland[12] calls attention to the strikingly beneficial, though temporary, effect of a high altitude in three cases of obstinate menorrhagia under his care, and he thinks it is a question whether residence at a high altitude should not be recommended in certain cases of chronic uterine hæmorrhage when ordinary methods of treatment, including curetting, are of no avail, and in which it is important to check the hæmorrhage in order to spare the patient the more serious operation of extirpation of the ovaries or of the uterus. There are many cases of uterine hæmorrhage in debilitated subjects, especially from fibroids, in which the risk of a major operation is dreaded by some surgeons as much as by the patient, and it is for such that Sunderland would advise residence at a high altitude. In selecting such a place, an elevated region with a low mean temperature should be chosen, where the air is as free from moisture as possible, and the soil well drained and dry, for in addition to the low temperature as a factor in the diminution of the flow, one must conclude that the dry, tonic, and bracing climate has a beneficial effect by improving the general condition of the patient. Possibly this bracing effect may also act through the nervous system, controlling and regulating menstruation. He thinks that the low pressure of the atmosphere at these altitudes may be an important factor in the prevention of certain hæmorrhages from the uterus, due possibly to the diminution in weight of the superimposed column of air on the surface of the abdomen. In the lungs it may act by lessening the pressure on the internal abdominal organs and large veins, thus making a more free flow of blood through the large veins and portal system, and diminishing uterine congestion.

[9] Med. Rec., Oct. 1, 1898, Charles Green Cumston. [10] Lancet, Oct. 1, 1898, F. J. McCann. [11] Lancet, Feb. 4, 1899, J. Inglis Parsons. [12] Lancet, Oct. 15, 1898, Septimus Sunderland.

